# Ancestral state reconstruction reveals multiple independent evolution of diagnostic morphological characters in the "Higher Oribatida" (Acari), conflicting with current classification schemes

**DOI:** 10.1186/1471-2148-10-246

**Published:** 2010-08-11

**Authors:** Sylvia Schäffer, Stephan Koblmüller, Tobias Pfingstl, Christian Sturmbauer, Günther Krisper

**Affiliations:** 1 Institute of Zoology, Karl-Franzens-University Graz, Universitätsplatz 2, 8010 Graz, Austria

## Abstract

**Background:**

The use of molecular genetic data in phylogenetic systematics has revolutionized this field of research in that several taxonomic groupings defined by traditional taxonomic approaches have been rejected by molecular data. The taxonomic classification of the oribatid mite group Circumdehiscentiae ("Higher Oribatida") is largely based on morphological characters and several different classification schemes, all based upon the validity of diagnostic morphological characters, have been proposed by various authors. The aims of this study were to test the appropriateness of the current taxonomic classification schemes for the Circumdehiscentiae and to trace the evolution of the main diagnostic traits (the four nymphal traits scalps, centrodorsal setae, sclerits and wrinkled cuticle plus octotaxic system and pteromorphs both in adults) on the basis of a molecular phylogenetic hypothesis by means of parsimony, likelihood and Bayesian approaches.

**Results:**

The molecular phylogeny based on three nuclear markers (28S rDNA, *ef-1α*, *hsp82*) revealed considerable discrepancies to the traditional classification of the five "circumdehiscent" subdivisions, suggesting paraphyly of the three families Scutoverticidae, Ameronothridae, Cymbaeremaeidae and also of the genus *Achipteria*. Ancestral state reconstructions of six common diagnostic characters and statistical evaluation of alternative phylogenetic hypotheses also partially rejected the current morphology-based classification and suggested multiple convergent evolution (both gain and loss) of some traits, after a period of rapid cladogenesis, rendering several subgroups paraphyletic.

**Conclusions:**

Phylogenetic studies revealed non-monophyly of three families and one genus as a result of a lack of adequate synapomorphic morphological characters, calling for further detailed investigations in a framework of integrative taxonomy. Character histories of six morphological traits indicate that their evolution followed a rather complex pattern of multiple independent gains (and losses). Thus, the observed pattern largely conflicts with current morphological classifications of the Circumdehiscentiae, suggesting that the current taxonomic classification schemes are not appropriate, apart from a recently proposed subdivision into 24 superfamilies.

## Background

Traditionally, morphological characters are the basis for taxonomy in the animal kingdom. In phylogenetic systematics derived or apomorphic characters are the working tools to reconstruct common ancestors which are further used for the grouping of taxa [1,2]. This concept differs from traditional systematics in that non-phylogenetic classifications are possibly artificial and not useful for asking evolutionary questions [3]. Henning's work was later popularized by the term "cladistics" which focuses on shared derived characters (synapomorphies). With help of these characters, cladistics aims at classifying species into monophyletic groups. Paraphyletic groups are therefore artificial and cannot be used within phylogenetic systematics [3]. Such artificial taxa are results of wrongly used synapomorphic characters caused by homoplastic character evolution and may mislead phylogenetic and taxonomic interpretations. In recent years, morphological analyses were often combined with molecular data to solve a variety of evolutionary and taxonomic problems [4-10]. This rise of combined analyses had an important impact on the nature of systematics [11]. Across many groups of animals, morphology-based classifications have been frequently revised based on new molecular phylogenies by identifying previously undetected homoplastic evolution of morphological characters (e.g. in the flatworm family Capsalidae [12], the genus *Dracus *[13], the lanternfly family Fulgoridae [14], or in Brazilian worm lizards [15]). Thus, after more than 250 years of predominance of comparative morphology in species discovery and taxonomic classification, future systematic and taxonomic research will, despite some skepticism [16], utilize combined evidence from both molecular and classical taxonomic approaches to enhance our understanding of biological systematics and to serve as basis for answering important questions in evolutionary biology research [11,17].

Traditionally, acarologists have used synapomorphic characters to classify mite species. However, based on this traditional taxonomic approach, phylogenetic relationships among many acarine taxa have remained unclear. Even the monophyly of the Acari is a matter of discussion [18], with two most recent studies providing strong evidence for a diphyly of the Acari [19,20]. Also within the two main lineages Anactinotrichida (= Opilioacariformes + Parasitiformes) and Actinotrichida (= Acariformes), some major classificatory changes have been made to reflect current concepts (see [21]). In Actionotrichida for example, recent studies based on either morphological, gland chemical or genetic data strongly indicate that the Astigmata represent a derived monophyletic group within the Oribatida [19,22,23], thus rendering the Oribatida paraphyletic. This case highlights the necessity of combining several analytic approaches to get insights into acarine systematics. Such comprehensive studies combining molecular genetic and morphological data are rare and the few examples are mainly addressing phylogenetic questions on family or genus level [10,24-26]. The phylogenetic relationships within the Oribatida are unclear, some studies solely based on molecular data addressed the phylogeography and/or phylogeny of selected taxa only [19,27-30] and very little emphasis has been put on characterizing the evolution of particular traits in a phylogenetic framework. Just one recent study [31] tested whether three particular traits, including two morphological characters, correlate with arboreal life-style in oribatid mites.

**Figure 1:**
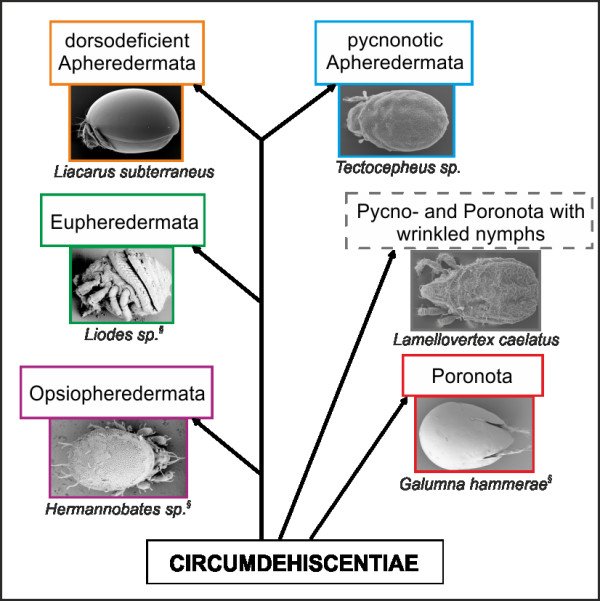
**The phylogenetic reconstruction of the Circumdehiscentiae based on 14 morphological characters modified after Wauthy**[37]. ^§ ^SEM micrographs modified from Hunt et al. [99].

This study is the first one to combine morphological and molecular genetic data to elucidate the phylogenetic relationships among the families assigned to the Circumdehiscentiae (= Brachypylina, "Higher Oribatida"), one of the six major groups of Oribatida [32]. The Circumdehiscentiae are the largest and taxonomically richest group of Oribatida, and although several studies aimed at resolving the taxonomy of this group, current taxonomic classifications within the Circumdehiscentiae are considered to be questionable [33]. Many controversial opinions regarding the use of morphological characters for diagnosing circumdehiscent taxa exist, since these characters might be subject to homoplastic evolution, thus not necessarily reflecting the phylogenetic relationships within the Circumdehiscentiae [34]. The first and most cited proposal regarding the systematics of the Circumdehiscentiae was set by Grandjean [35] who defined five subdivisions based on three main characters: i) scalps in nymphs. A scalp is a part of the exuvia of the gastronotic region that is retained on an emerging nymph (or adult in some genera) after the moult. Grandjean called nymphs that retain scalps as eupheredermous; nymphs that do not as apheredermous. In one family (Hermanniellidae) nymphs do not retain scalps but the adults possess the tritonymphal scalp; this characteristic is called opsiopheredermous. One further exception concerns the Oribatellidae: here the species are apopheredermous, with nymphs retaining scalps which are held away from the body by setae. ii) three pairs of centrodorsal setae *da*, *dm *and *dp*; if these setae are lost in nymphs this trait is called dorsodeficient, if setae are present integridorsal. iii) the octotaxic system in adult mites. This system is a special series of originally four pairs of secretory [36] notogastral porose organs (developed as porose areas or saccules) which can vary in size, shape and number. Species featuring the octotaxic system are called poronotic, species without it pycnonotic. Based on these three characters Grandjean defined the five subdivisions Opsiopheredermata, Eupheredermata, dorsodeficient Apheredermata, normal pycnonotic (= integridorsal) Apheredermata and Poronota. Within the Poronota Grandjean [35] formulated three groups according to the appearance of nymphs and larvae (either wrinkled or smooth with micro- or macrosclerits). This classification was also adopted by Wauthy [37] who included a sixth group, the "Circumdehiscentiae (= Pycno- and Poronota) with wrinkled nymphs" to the Circumdehiscentiae, based on ideas of Grandjean [35,38]. In these works, Grandjean assumes that eleven families, including taxa of Eu- and Apheredermata which have nymphs with a wrinkled gastronotic cuticle structure form a monophyletic entity. The phylogenetic relationships among the six subdivisions based on a parsimony analysis of 14 morphological characters are shown in Fig. 1 (modified from Wauthy [37]). Please note that Wauthy's results have never been formally published, but since this study represents the only available cladistic analysis of the Circumdehiscentiae based on morphological characters, we decided to adopt his classification scheme for our analyses. Recent hypotheses mostly avoid Grandjean's old classification scheme. For example, Subías [39] simply classified the Circumdehiscentiae into two groups, the "Pycno- and Poronoticae" and Norton and Behan-Pelletier [34] presented a system without any higher grouping of the taxa, but assigning the circumdehiscent taxa to 24 superfamilies.

To evaluate which of these classifications is the most appropriate one for the Circumdehiscentiae, we inferred a molecular phylogeny and traced several diagnostic morphological characters on the phylogeny. Following Grandjean's [35] classification, we investigated the three main traits (scalps, centrodorsal setae, octotaxic system) plus three additional characters which are also commonly used for categorization: the presence of micro- and macrosclerits in nymphs, the wrinkled cuticle structure of nymphs, and furthermore the development of so-called pteromorphs in adults. Pteromorphs are humeral projections on the lateral border of the notogaster, concealing all or parts of the adducted legs. In some taxa these are immotile structures, whereas in other taxa the base of pteromorphs is de-sclerotized, forming a linear hinge, and inserted with a highly differentiated musculature, allowing for motility of this structure. This trait is known from many poronotic mites and might potentially serve as synapmorphic character within this group.

The aims of the present study are i) evaluating the validity of Grandjean's [35] and Wauthy's [37] taxonomic classification of the Circumdehiscentiae; ii) tracing the evolution of five diagnostic traits (nymphal scalps, centrodorsal setae, sclerits and wrinkled cuticle plus octotaxic system in adults) commonly used for taxonomic classification of circumdehiscent mites; and iii) investigating the potential of pteromorphs for categorization within the Poronota. To achieve our goals, we established a molecular phylogeny based on three nuclear markers, 28S ribosomal DNA, elongation factor-1alpha (*ef-1α*) and heat shock protein 82 (*hsp82*), that are commonly used for phylogenetic studies in arthropods [40-44] and in particular for phylogenetic inference in mites, especially in Oribatida [26,27,29,45-47], and traced the evolution of our six morphological traits of interest over the molecular phylogeny using parsimony, likelihood and Bayesian approaches.

## Methods

### Sample collection

This study includes 40 representatives of all five subdivisions of Circumdehiscentiae (after Grandjean [35]): Opsiopheredermata, Eupheredermata, dorsodeficient and pycnonotic Apheredermata and Poronota (Table 1). Families categorized as the sixth subdivision "Circumdehiscentiae with wrinkled nymphs" (after Wauthy [37]) are written in bold lettering in the table. Based on Weigmann [48] we chose *Hermannia gibba *(Hermanniidae), a member of the Desmonomata, sister group of the Circumdehiscentiae, as outgroup. Sequences not generated in the framework of this study were obtained from GenBank (see Table 1).

**Table 1:** Specimens, GenBank accession numbers and coding of morphological characters for the analyzed samples.

Classification	Species	**GenBank Accession No**.			sca	cd	os	scl	gc	pt
								
		28S	*ef-1α*	hsp82						
OUTGROUP										
Hermanniidae										
*Hermannia*	*gibba*	AY273530^a^	EF081327^a^	DQ090800^a^	0	0	0	0	1	0
	*gibba*	HM582379	HM582404	HM582356	0	0	0	0	1	0
OPSIOPHEREDERMATA										
Hermanniellidae										
*Hermanniella*	*punctulata*	HM582381	HM582406	HM582358	1	0	0	0	0	0
EUPHEREDERMATA										
Neoliodidae										
*Platyliodes*	*scaliger*	HM582376	HM582401	HM582353	2	2	0	0	0	0
	*sp.*	HM582375	HM582400	HM582352	2	2	0	0	0	0
Gymnodamaeidae										
*Arthrodamaeus*	*sp.*	HM582387	HM582412	---	2	2	0	0	0	0
Eutegaeidae										
*Eutegaeus*	*curviseta*	DQ090816^a^	EF081326^a^	DQ090789^a^	2	2	0	0	0	0
Zetorchestidae										
*Zetorchestes*	*falzonii*	HM582386	HM582411	HM582363	2	2	0	0	0	0
Niphocepheidae										
*Niphocepheus*	*nivalis*	HM582377	HM582402	HM582354	2	2	0	0	0	0
DORSODEFICIENT APHEREDERMATA										
Liacaridae										
*Liacarus*	*cf. subterraneus*	HM582389	HM582413	---	2	2	0	0	0	0
Peloppiidae										
*Ceratoppia*	*quadridentata*	HM582378	HM582403	HM582355	2	2	0	0	0	0
NORMAL PYCNONOTIC APHEREDERMATA										
Carabodidae										
*Carabodes*	*femoralis*	AY273508^a^	EF081325^a^	DQ090786^a^	3	1	0	0	0	0
	*labyrinthicus*	AY273506^a^	EF093762^a^	EF093765^a^	3	1	0	0	0	0
	*marginatus*	HM582382	HM582407	HM582359	3	1	0	0	0	0
Hydrozetidae										
*Hydrozetes*	*lacustris*	HM582370	HM582395	HM582347	3	1	0	0	0	0
	*lemnae*	HM582369	HM582394	HM582346	3	1	0	0	0	0
**Tectocepheidae**										
*Tectocepheus*	*velatus*	EF093757^a^	EF093763^a^	EF093770^a^	3	1	0	0	1	0
	*minor*	EF093756^a^	EF093764^a^	EF093772^a^	3	1	0	0	1	0
	*sarekensis*	EF093759^a^	EF093760^a^	EF093774^a^	3	1	0	0	1	0
	*cf. alatus*	HM582383	HM582408	HM582360	3	1	0	0	1	0
**Ameronothridae**										
*Ameronothrus*	*maculatus*	HM582372	HM582397	HM582349	3	1	0	0	1	0
*Podacarus*	*auberti cf. occidentalis*	HM582373	HM582398	HM582350	3	1	0	0	1	0
**Cymbaeremaeidae**										
*Cymbaeremaeus*	*cymba*	GU208575^a^	GU208670^a^	HM582340	3	1	0	0	1	0
*Scapheremaeus*	*cf. palustris*	HM582371	HM582396	HM582348	3	1	0	0	1	0
**Ametroproctidae**										
*Ametroproctus*	*lamellatus*	HM582364	HM582389	HM582341	3	1	0	0	1	0
PORONOTA										
Galumnidae										
*Galumna*	*cf. obvia*	HM582368	HM582393	HM582345	3	1	1	2	0	2
Ceratozetidae										
*Trichoribates*	*trimaculatus*	HM582384	HM582409	HM582361	3	1	1	2	0	1
Euzetidae										
*Euzetes*	*globulus*	HM582374	HM582399	HM582351	3	1	1	2	0	1
Oribatulidae										
*Phauloppia*	*cf. lucorum*	HM582385	HM582410	HM582362	3	1	1	1	0	0
**Scutoverticidae**										
*Scutovertex*	*minutus*	GU208538^a^	GU208633^a^	HM582332	3	1	3	0	1	0
	*sculptus*	GU208550^a^	GU208645^a^	HM582333	3	1	3	0	1	0
	*pannonicus*	GU208540^a^	GU208635^a^	HM582334	3	1	3	0	1	0
	*pileatus*	GU208544^a^	GU208639^a^	HM582336	3	1	3	0	1	0
*"Scutovertex"*	*pictus*	GU208541^a^	GU208636^a^	HM582335	3	1	0	0	1	0
*Provertex*	*kuehnelti*	GU208567^a^	GU208662^a^	HM582339	3	1	0	0	1	0
*Lamellovertex*	*caelatus*	GU208565^a^	GU208660^a^	HM582337	3	1	0	0	1	0
*Exochocepheus*	*hungaricus*	GU208570^a^	GU208665^a^	HM582338	3	1	3	0	1	0
**Phenopelopidae**										
*Eupelops*	*cf. curtipilus*	HM582380	HM582405	HM582357	3	1	1	0	1	2
**Unduloribatidae**										
*Unduloribates*	*undulatus*	HM582365	HM582390	HM582342	3	1	0	0	1	1
**Achipteriidae**										
*Parachipteria*	*punctata*	HM582366	HM582391	HM582343	3	1	1	0	1	1
*Achipteria*	*coleoptrata*	AY273500^a^	AY632776^a^	EF081335^a^	3	1	2	0	1	1
	*quadridentata*	HM582367	HM582392	HM582344	3	1	2	0	1	1

Families written in bold lettering are assigned to the subdivision "Circumdehiscentiae with wrinkled nymphs" after [23]. ^a ^= Sequences obtained from GenBank.

Morphological characters and character coding used in this study: sca, scalps (0 = outgroup, 1 = opsiopheredermous, 2 = eupheredermous, 3 = apheredermous); cd, centrodorsal setae (0 = holotrich, 1 = integridorsal, 2 = dorsodeficient); os, octotaxic system (0 = no porose organs, 1 = porose areas, 2 = saccules type1, 3 = saccules type2); scl, sclerits (0 = nymphs nude, 1 = nymphs with microsclerits, 2 = nymphs with macrosclerits); gc, gastronotic cuticle of nymphs (0 = unwrinkled, 1 = wrinkled); pt, pteromorphs (0 = no pt, 1 = pt immotile, 2 = pt motile).

Specimens were extracted from mosses, lichens or soil samples with Berlese-Tullgren funnels and preserved in absolute ethanol. Total genomic DNA was extracted from single individuals applying the CTAB (hexadecyltriethylammonium bromide) method described in Schäffer et al. [30]. After DNA extraction, the sclerotized body remnants were mounted on permanent slides and used for species identification using the criteria defined in Weigmann [33].

### PCR and DNA sequencing

Fragments of 28S rDNA, *ef-1α *and *hsp82 *genes were amplified by polymerase chain reaction (PCR) using the following primers: D3A and D3B [49] for the D3 fragment of the 28S rDNA, 40.71F and 52.RC [50] and EF-SyFwd and EF-SyRev [10] for *ef-1α*, hsp1.2 and hsp8.x [42] for *hsp82*. Polymerase Chain Reaction (PCR), purification of PCR products and DNA sequencing followed the protocol described in Schäffer et al. [51]. DNA fragments were purified with Sephadex™ G-50 (Amersham Biosciences) following the manufacturer's instruction and sequencing reaction products were analyzed on an ABI PRISM 3130xl automated sequencer (Applied Biosystems). Sequences are available from GenBank under the accession numbers listed in Table 1.

### Alignment and phylogenetic analyses

We sequenced 311-329 bp of the D3 region of the 28S rDNA, 475 bp of the *ef-1α*, and 467-503 bp of the *hsp82 *gene in 34 specimens (from *Liacarus cf. subterraneus *and *Arthrodamaeus sp*. the fragment of *hsp82 *could not be amplified). Sequences were verified by comparisons with known oribatid sequences from GenBank and aligned by eye in MEGA 3.1 [52]. We removed poorly aligned regions from the alignments of 28S rDNA and *hsp82 *using the program trimAl [53] which is a tool for automated alignment trimming. Gap threshold was set to 0.8 and similarity threshold to 0.001. All sequences were combined into a single data set with a resulting length of 1,298 bp for further analyses.

Phylogenetic inference was based on Bayesian inference (BI), conducted in MrBayes 3.1.2 [54]. Data were partitioned by gene and the *ef-1α *gene was further partitioned by codon position. Number of substitution types was set to six (GTR model) for each data partition and among-site rate variation was drawn from a gamma distribution. Posterior probabilities were obtained from a Metropolis-coupled Markov chain Monte Carlo simulation (2 independent runs; 4 chains with two million generations each; trees sampled every 100 generations), with parameters estimated from the data set. Mixing and convergence to stationary distributions were evaluated in Tracer v.1.4 by inspecting graphically the trace of the parameter against the generation numbers [55]. The first 4000 (20%) trees were discarded as burn-in prior to constructing a 50% majority-rule consensus from the remaining 16,001 trees.

### Analyses of character evolution

Ancestral state reconstruction (ASR) is an increasingly popular method to map morphological or ecological traits onto a molecular phylogeny. However, there are still controversial opinions about the accuracy of commonly used methods (maximum parsimony (MP), maximum likelihood (ML), Bayesian) and each suffers from certain advantages and limitations [56-59]. According to Ekman et al. [60], who showed the importance of conducting ASR with more than one method, we performed our analyses using all three above mentioned methods. Furthermore, specifying the right models and priors in a Bayesian analysis is of uttermost importance [60,61]. We employed the reversible-jump (RJ) MCMC, where models are visited in proportion to their posterior probability [62].

**Figure 2:**
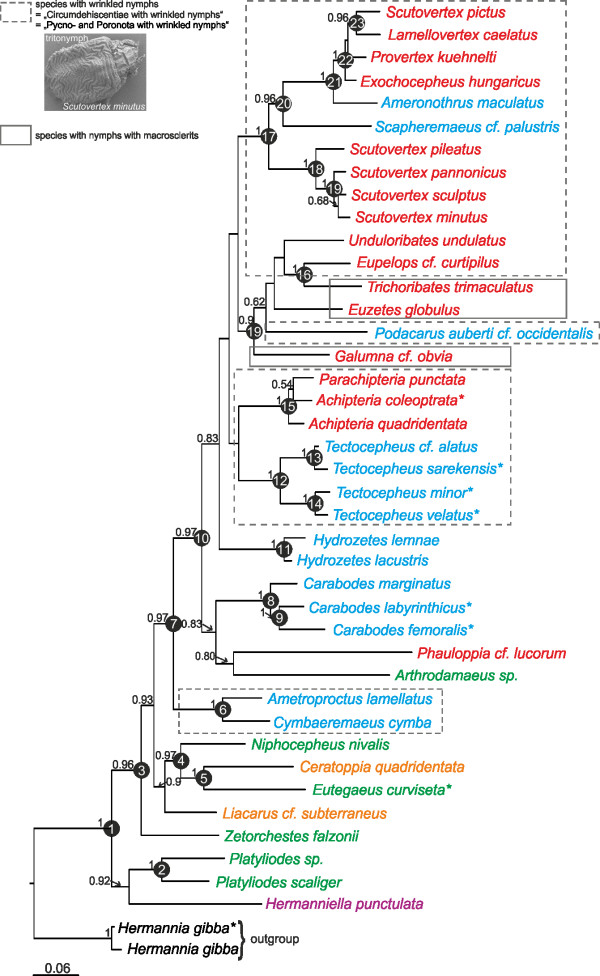
**Bayesian 50% majority rule consensus tree of 40 representatives of all five subdivisions of Circumdehiscentiae **[35]**: Opsiopheredermata (violet), Eupheredermata (green), dorsodeficient Apheredermata (orange), pycnonotic Apheredermata (blue) and Poronota (red)**. The tree is based on a combined data set of all available fragments of the 28S rDNA, *ef-1α *and *hsp82 *genes. Posterior probabilities >50 are shown. Numbers at nodes indicate nodes that have been used to assess ancestral states. *Sequence data of taxon obtained from GenBank.

We traced the evolution of six morphological characters (scalps, centrodorsal setae, sclerits and wrinkled cuticle which are all developed in nymphs plus octotaxic system and pteromorphs both in adults; Table 1) over the molecular phylogeny using MP, ML and Bayesian approaches. Information on the studied characters was retrieved from the literature [33,35,51,63-71] and from the body remnants of our specimens. Ancestral character state reconstruction for the notogastral octotaxic system, employed two different data sets, one (titled porose organs-1) with porose organs (regardless of which type) as either absent (0) or present (1) and one (titled porose organs-2) coded with the different types found in oribatid mites: porose organs absent (0), porose areas (1), saccules type1 (2), saccules type2 (3). With reference to Alberti et al [36] saccules type2 differs from type1 by lacking an elaborate microvilli system, having a considerable number of mitochondria and rather characteristic lysosome-like inclusions. Parsimony and likelihood based ASR were conducted in Mesquite v.2.71 [72]. To account for topological uncertainty we used the "trace character over trees" option, which summarizes the ASR over a series of trees. All reconstructions were integrated over the last 6001 post burn-in trees of the Bayesian analysis and the ancestral states were summarized on the BI consensus tree. As model of evolution for the ML reconstructions we employed the Markov k-state 1 (Mk1) parameter model, with equal probability for any particular character change. To account for phylogenetic mapping uncertainty, we further evaluated probabilities of ancestral states calculated from the same 6001 BI trees using the MCMC method in BayesMultiState [73], implemented in the BayesTraits 1.0 package. Ancestral states were only reconstructed for 23 nodes (see Fig. 2), which were selected based on their posterior probability support values of the BI analysis (only those nodes with PP ≥ 0.95 were used). A reversible-jump (RJ) hyperprior with a gamma prior (exponential prior seeded from a uniform distribution on the interval 0 to 30) was used to reduce uncertainty and arbitrariness of choosing priors in the MCMC analysis. According to preliminary analyses, we set the *ratedev *value to 8, achieving an acceptance rate of proposed changes between 20 and 40%. The option "AddNode" was used to find the proportion of the likelihood associated with each of the possible states at each node. Three independent MCMC runs were performed with 5,050,000 iterations. Chains were sampled every 100 iteration after a burn-in of 50,000 iterations. Because of similar results of the three runs, we only report one of them here. The output files were analyzed using Tracer v1.4.

### Testing alternative hypothesis of character evolution

Bayes factors (BF) are statistical tools to compare alternative hypotheses against a null hypothesis [74-76]. We used BF to test ten alternative phylogenetic hypotheses against the unconstrained BI tree: Monophyly of i) Apheredermata (hypothesis 1), ii) the dorsodeficient nymphs (hypothesis 2), iii) Poronota (hypothesis 3), iv) nymphs with macrosclerits (hypothesis 4), v) "Circumdehiscentiae with wrinkled nymphs" (hypothesis 5), and vi) pteromorphs (hypothesis 6). Furthermore, we tested the monophyly of the families Scutoverticidae (hypothesis 7), Cymbaeremaeidae (hypothesis 8) and Ameronothridae (hypothesis 9), plus monophyly of each of the three families (hypothesis 10). Alternative phylogenetic trees were inferred in MrBayes by applying topological constraints. Run settings were the same as for the unconstrained BI tree. We combined the two log-files of the Bayesian analysis with the program LogCombiner v1.5.4 available in BEAST package [77] and calculated the BF in Tracer. Standard errors were assessed using 1,000 bootstrap replicates. Interpretation of BF followed Kass and Raftery [76].

## Results

### Phylogenetic analysis

Pairwise sequence divergence (uncorrected p-distances) between the investigated species ranged from 0-11% in the D3 fragment of 28S rDNA, from 0-22% in the *ef-1α *gene and from 2-26% in the *hsp82 *gene. In the combined data set, pairwise differences ranged from 1-20%.

The Bayesian 50% majority rule consensus tree is shown in Fig. 2. Most basal nodes were statistically well supported whereas some more recent splits were poorly resolved. Compared to the traditional classification, our BI tree revealed some discrepancies. None of the five or six, respectively, major subdivisions seems to be monophyletic. In particular, the "Eupheredermata", "dorsodeficient Apheredermata" and "Poronota" appeared as para- or polyphyletic. Wauthy's [37] subdivision "Circumdehiscentiae with wrinkled nymphs" clusters together in a major clade, but also includes three species of Poronota (*Trichoribates trimaculatus*, *Euzetes globulus *and *Galumna cf. obvia*), though with low statistical support. The two "circumdehiscent" species *Cymbaeremaeus cymba *and *Ametroproctus lamellatus *form a well supported more basal clade, rendering the "Circumdehiscentiae with wrinkled nymphs" paraphyletic. Likewise, the family Scutoverticidae was not resolved as monophylum, but rather constitutes two distinct clusters: one includes exclusively species of the genus *Scutovertex*, whereas the other one comprises members of the three other genera - *Provertex kuehnelti*, *Lamellovertex caelatus*, *Exochocepheus hungaricus - *plus "*Scutovertex pictus*" and furthermore *Ameronothrus maculatus *and *Scapheremaeus cf. palustris*, thus rendering the family paraphyletic. BF of the tested alternative hypothesis rejected a monophyletic family Scutoverticidae (hypothesis 7, Table 2). The aforementioned results also imply paraphyly of the families Cymbaeremaeidae and Ameronothridae supported by the BF which decisively discriminated against a monophyly of the two families (hypotheses 8 and 9, Table 2). Also the monophyly of each of the three families was rejected by the BF (hypothesis 10, Table 2). Moreover, *Achipteria coleoptrata *clusters with *Parachipteria punctata *and *Achipteria quadridentata *as sister taxon, also rendering the genus *Achipteria *paraphyletic; alternatively, *Parachipteria *might represent a synonym of *Achipteria*.

**Table 2:** Comparison of alternative phylogenetic hypotheses against the unconstrained Bayesian inference (BI) tree using Bayes factors.

Trace	ln P (model/data)	Standard error	log10 Bayes Factors	Interpretation Bayes Factors
BI	-15019,465	± 0,123	-	
hypothesis 1	-15029,989	± 0,125	-4,57	Decisive
				
BI	-15019,465	± 0,125	-	
hypothesis 2	-15065,923	± 0,124	-20,176	Decisive
				
BI	-15019,465	± 0,12	-	
hypothesis 3	-15177,437	± 0,112	-68,606	Decisive
				
BI	-15019,465	± 0,121	-	
hypothesis 4	-15039,901	± 0,117	-8,875	Decisive
				
BI	-15019,465	± 0,117	-	
hypothesis 5	-15113,273	± 0,119	-40,74	Decisive
				
BI	-15019,465	± 0,12	-	
hypothesis 6	-15023,101	± 0,125	-1,579	Strong
				
BI	-15019,465	± 0,12	-	
hypothesis 7	-15091,084	± 0,112	-31,104	Decisive
				
BI	-15019,465	± 0,119	-	
hypothesis 8	-15073,085	± 0,121	-23,287	Decisive
				
BI	-15019,465	± 0,12	-	
hypothesis 9	-15127,044	± 0,114	-46,721	Decisive
				
BI	-15019,465	± 0,122	-	
hypothesis 10	-15197,565	± 0,113	-77,348	Decisive

### Ancestral state reconstruction

**Figure 3:**
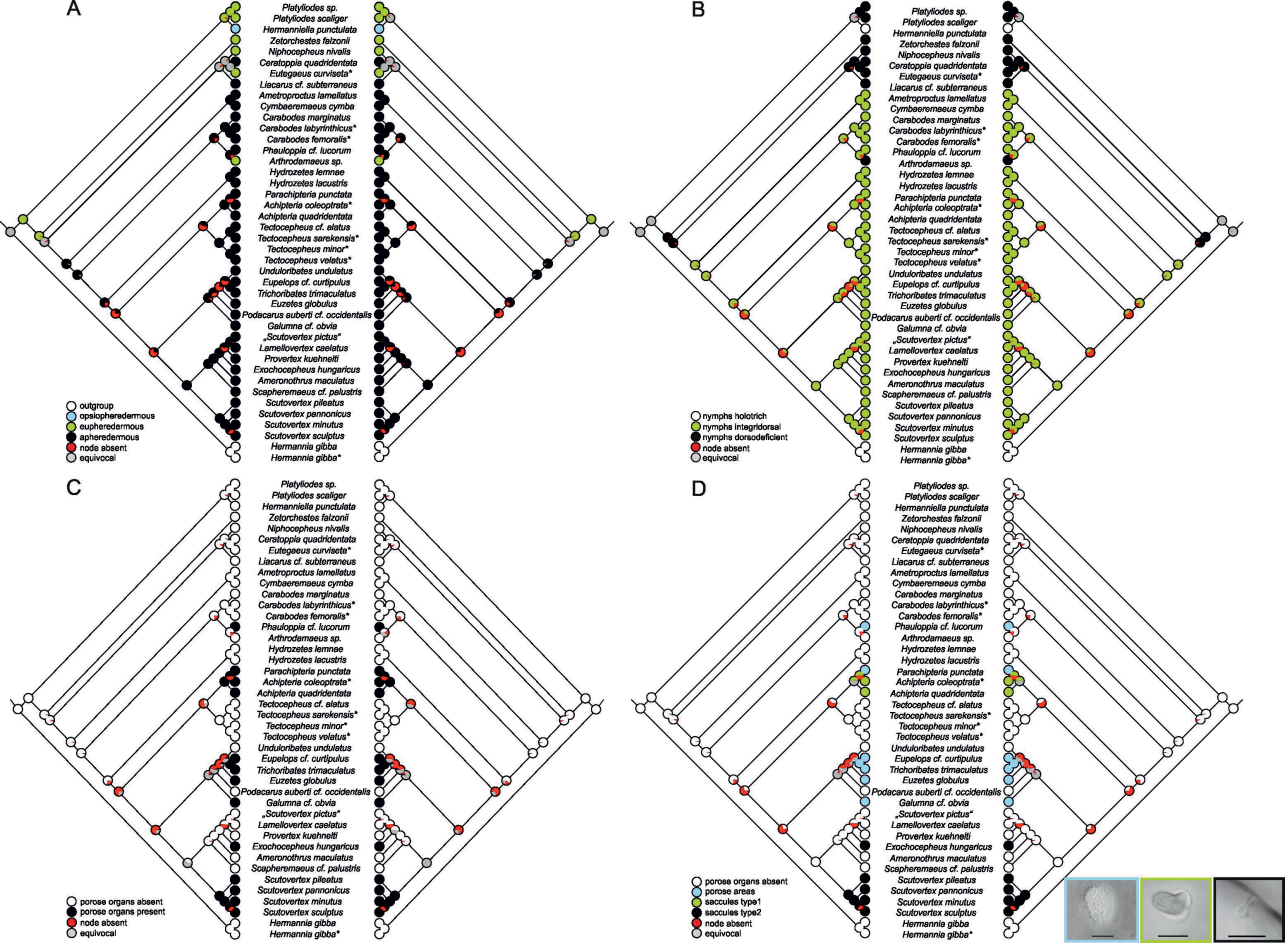
**Ancestral state reconstructions for the Circumdehiscentiae based on parsimony (left cladogram) and likelihood (right cladogram) of (A) scalps in nymphs, (B) centrodorsal setae in nymphs, (C) porose organs (two-stated character coding), and (D) the different types of porose organs found in oribatid mites**. Light micrographs with differential interference contrast showing one porose area of *Trichoribates trimaculatus*, one saccule type1 of *Achipteria coleoptrata *and one saccule type2 of *Scutovertex pannonicus *(from left to right). Scale bars: 10 μm. *Sequence data of taxon obtained from GenBank.

**Figure 4:**
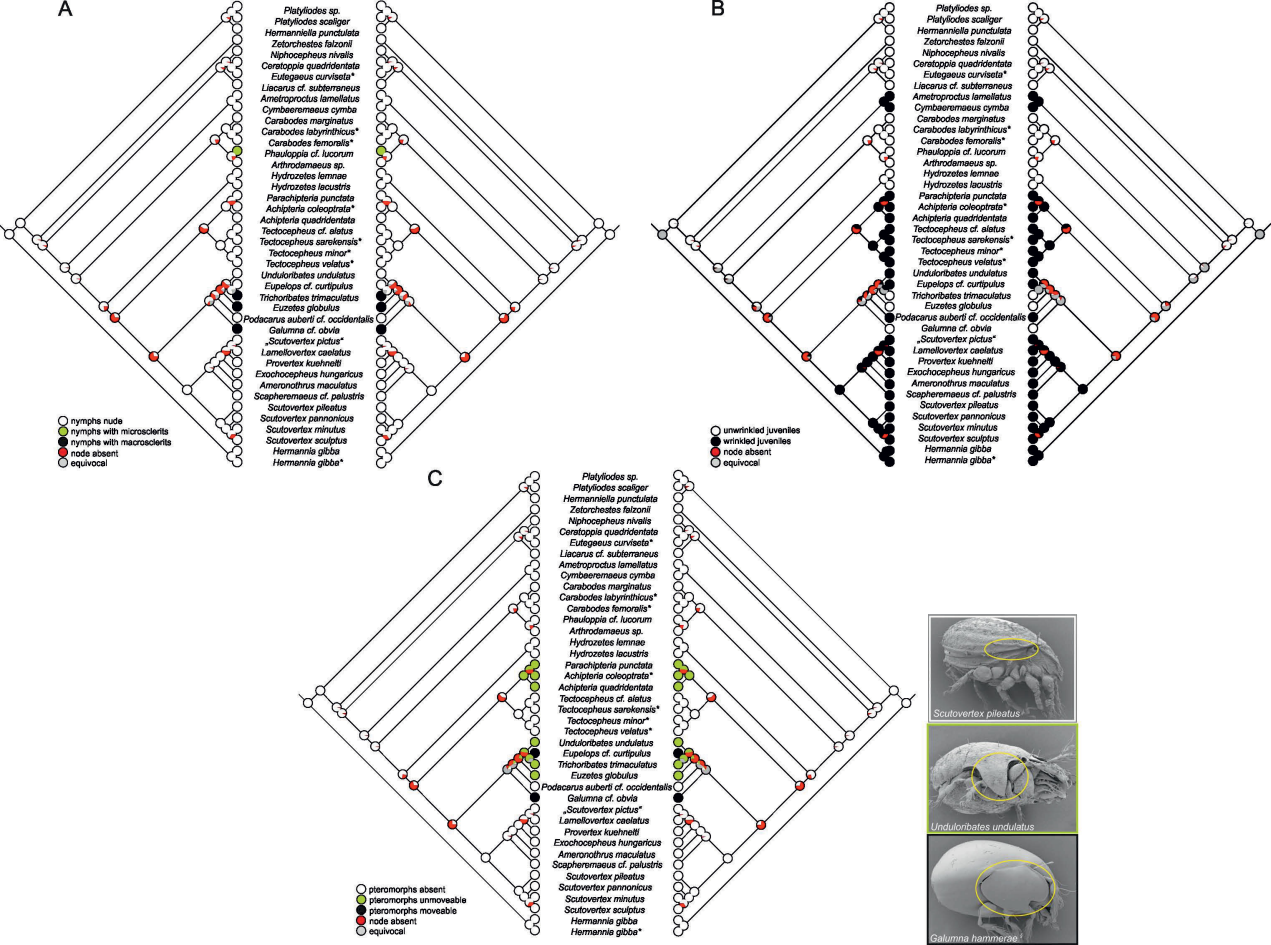
**Ancestral state reconstructions for the Circumdehiscentiae based on parsimony (left cladogram) and likelihood (right cladogram) of (A) sclerits in nymphs, (B) wrinkles in nymphs, and (C) pteromorphs in adults**. ^§ ^SEM micrographs modified from Hunt et al. [99]. *Sequence data of taxon obtained from GenBank.

The results from the ASR of scalps, centrodorsal setae, porose organs, sclerits in nymphs, nymphal cuticle structure and pteromorphs are shown in Figs. 3, 4 and Table 3. The reconstruction yielded no conflicts between parsimony, likelihood and Bayesian analyses except for some nodes, depending on the studied character (Table 3), but the likelihood approach reconstructed them with greater uncertainty (equivocal) than the Bayesian approach. For example, nodes 4 and 5 in scalp evolution (Fig. 3A) or node 17 in data set porose organs-1 (Fig. 3C) were ambiguously reconstructed in the likelihood analysis as compared to the Bayesian analysis (Table 3). On the other hand, the likelihood analysis reconstructed some nodes with greater certainty for a particular character state than the Bayesian approach, as for example nodes 1 and 3 in scalp evolution (Fig. 3A, Table 3) or node 20 in the reconstruction of gastronotic cuticle structure evolution (Fig. 4B, Table 3).

**Table 3:** Mean of posterior probabilities (PPs) of the Bayesian reconstruction for the ancestral states of six studied characters at 23 nodes from Fig. 2.

	scalps				centrodorsal setae			porose organ - 1		porose organ -2			
	
node	0	1	2	3	0	1	2	0	1	0	1	2	3
**1**	0.129	0.161	0.551	0.159	0.193	1.192E-2	0.795	**0.972**	2.842E-2	0.823	4.625E-2	5.619E-2	7.422E-2
**2**	7.195E-2	6.589E-2	0.839	2.302E-2	4.564E-2	3.798E-3	**0.951**	**0.959**	4.053E-2	0.815	4.824E-2	5.965E-2	7.698E-2
**3**	8.732E-2	8.214E-2	0.283	0.548	7.921E-2	3.625E-2	0.885	**0.977**	2.298E-2	0.822	5.025E-2	5.614E-2	7.118E-2
**4**	0.114	0.102	0.619	0.165	5.281E-2	3.741E-3	**0.943**	**0.948**	5.189E-2	0.783	5.799E-2	6.899E-2	9.014E-2
**5**	0.138	0.125	0.338	0.399	8.779E-2	1.109E-2	**0.901**	**0.907**	9.299E-2	0.706	8.391E-2	9.421E-2	0.116
**6**	3.41E-2	3.582E-2	2.456E-2	**0.906**	1.762E-2	**0.974**	8.361E-3	**0.972**	2.752E-2	0.854	3.664E-2	4.744E-2	6.183E-2
**7**	4.212E-2	4.355E-2	3.752E-2	0.877	0.186	0.573	0.241	**0.936**	6.441E-2	0.638	0.114	0.129	0.12
**8**	1.541E-2	1.75E-2	7.441E-3	**0.96**	6.358E-3	**0.992**	1.756E-3	**0.992**	7.518E-3	**0.933**	1.491E-2	2.171E-2	3.002E-2
**9**	2.793E-2	2.776E-2	1.686E-2	**0.927**	1.411E-2	**0.981**	5.184E-3	**0.982**	1.764E-2	0.89	2.761E-2	3.477E-2	4.764E-2
**10**	3.423E-2	3.736E-2	3.452E-2	0.894	0.173	0.648	0.178	0.861	0.139	0.525	0.163	0.179	0.134
**11**	7.894E-3	8.032E-3	3.046E-3	**0.981**	3.285E-3	**0.996**	7.709E-4	**0.997**	2.789E-3	**0.973**	5.943E-3	8.732E-3	1.209E-2
**12**	3.029E-2	3.006E-2	1.842E-2	**0.921**	1.524E-2	**0.979**	5.591E-3	**0.981**	1.927E-2	0.88	3.016E-2	3.778E-2	5.183E-2
**13**	3.394E-3	3.491E-3	1.068E-3	**0.992**	1.259E-3	**0.998**	2.525E-4	**0.999**	9.317E-4	**0.99**	2.211E-3	3.451E-3	4.733E-3
**14**	9.165E-3	9.32E-3	3.52E-3	**0.978**	3.799E-3	**0.995**	8.827E-4	**0.997**	3.238E-3	**0.969**	6.899E-3	1.013E-2	1.398E-2
**15**	8.217E-3	8.786E-3	2.644E-3	**0.98**	2.728E-3	**0.997**	5.34E-4	2.432E-3	**0.998**	**0.97**	6.732E-3	1.016E-2	1.351E-2
**16**	4.279E-2	4.315E-2	3.287E-2	0.881	2.16E-2	**0.967**	1.17E-2	4.278E-2	**0.957**	1.322E-2	0.811	0.115	6.12E-2
**17**	3.657E-2	3.75E-2	2.507E-2	**0.901**	1.539E-2	**0.978**	6.504E-3	0.705	0.295	0.447	6.018E-2	9.243E-2	0.4
**18**	1.779E-2	1.881E-2	9.119E-3	**0.954**	7.636E-3	**0.99**	2.581E-3	1.13E-3	**0.999**	5.012E-3	1.281E-2	2.444E-2	**0.958**
**19**	4.386E-3	4.75E-3	1.285E-3	**0.99**	1.501E-3	**0.998**	2.755E-4	1.146E-3	**0.999**	3.938E-4	2.133E-3	4.276E-3	**0.993**
**20**	5.455E-2	5.458E-2	4.692E-2	0.844	3.079E-2	**0.951**	1.817E-2	**0.935**	6.511E-2	0.716	6.596E-2	7.736E-2	0.141
**21**	1.577E-2	1.882E-2	8.806E-3	**0.958**	7.628E-3	**0.99**	2.482E-3	**0.964**	3.612E-2	0.751	2.409E-2	3.333E-2	0.191
**22**	4.429E-3	5.474E-2	1.244E-3	**0.989**	1.487E-3	**0.998**	1.93E-4	0.887	0.113	0.639	1.725E-2	2.59E-2	0.318
**23**	1.679E-2	1.884E-2	9.5E-3	**0.955**	8.235E-3	**0.989**	2.654E-3	**0.991**	9.344E-3	**0.933**	1.609E-2	2.144E-2	2.975E-2
				
	**sclerits**			**wrinkles**			**pteromorphs**						
						
**node**	**0**	**1**	**2**	**0**	**1**	**0**	**1**	**2**					
					
**1**	**0.933**	3.084E-2	3.575E-2	0.702	0.298	**0.964**	1.501E-2	2.146E-2					
**2**	**0.927**	3.285E-2	3.984E-2	**0.944**	5.638E-2	**0.953**	2.034E-2	2.687E-2					
**3**	**0.942**	2.562E-2	3.196E-2	0.679	0.321	**0.954**	1.902E-2	2.702E-2					
**4**	**0.919**	3.707E-2	4.428E-2	**0.929**	7.101E-2	**0.947**	2.316E-2	3.032E-2					
**5**	0.89	4.856E-2	6.167E-2	0.871	0.129	**0.91**	4.249E-2	4.722E-2					
**6**	**0.939**	2.765E-2	3.293E-2	2.422E-2	**0.976**	**0.965**	1.43E-2	2.036E-2					
**7**	**0.92**	3.53E-2	4.471E-2	0.214	0.786	0.888	5.379E-2	5.85E-2					
**8**	**0.964**	1.673E-2	1.898E-2	**0.99**	9.715E-3	**0.988**	4.413E-3	8.04E-3					
**9**	**0.955**	2.016E-2	2.467E-2	**0.975**	2.473E-2	**0.97**	1.126E-2	1.852E-2					
**10**	**0.904**	4.18E-2	5.375E-2	0.445	0.555	0.843	8.328E-2	7.343E-2					
**11**	**0.985**	6.909E-3	8.138E-3	**0.996**	3.826E-3	**0.994**	2.024E-3	4.461E-3					
**12**	**0.952**	2.177E-2	2.664E-2	1.737E-2	**0.983**	**0.968**	1.235E-2	2.006E-2					
**13**	**0.993**	3.313E-3	3.835E-3	7.777E-4	**0.999**	**0.998**	7.118E-4	1.772E-3					
**14**	**0.983**	8.015E-3	9.392E-3	2.538E-3	**0.997**	**0.992**	2.346E-3	5.213E-3					
**15**	**0.981**	9.517E-3	9.287E-3	1.672E-3	**0.998**	2.801E-4	**0.988**	1.144E-2					
**16**	0.622	5.949E-2	0.318	0.312	0.688	8.554E-3	0.534	0.454					
**17**	**0.938**	2.91E-2	3.278E-2	2.378E-2	**0.976**	**0.96**	1.591E-2	2.411E-2					
**18**	**0.967**	1.627E-2	1.722E-2	6.856E-3	**0.993**	**0.984**	5.671E-3	1.028E-2					
**19**	**0.989**	5.689E-3	5.302E-3	7.406E-4	**0.999**	**0.997**	7.603E-4	1.839E-3					
**20**	**0.915**	3.907E-2	4.572E-2	4.787E-2	**0.952**	**0.936**	2.884E-2	3.511E-2					
**21**	**0.966**	1.663E-2	1.757E-2	6.997E-3	**0.993**	**0.986**	5.335E-3	8.978E-3					
**22**	**0.985**	7.372E-3	7.43E-3	6.712E-4	**0.999**	**0.997**	7.688E-4	1.837E-3					
**23**	**0.965**	1.696E-2	1.809E-2	7.417E-3	**0.993**	**0.984**	5.782E-3	9.748E-3					

Bold numbers indicate PPs greater than 0.90.

The reconstruction of character evolution of the nymphal scalps (Fig. 3A) and centrodorsal setae (Fig. 3B) revealed a more or less single origin of the different character states, with the exceptions of the two apheredermous species *Ceratoppia quadridentata *and *Liacarus cf. subterraneus*, which clustered together with eupheredermous species, and the eupheredermous *Arthrodamaeus sp.*, which grouped with apheredermous species. The phylogenetic placement of the latter species implies that dorsodeficient nymphs evolved twice. BF of alternative hypotheses reject both the monophyly of Apheredermata (hypothesis 1, Table 2) and a monophyletic origin of dorsodeficient nymphs (hypothesis 2, Table 2). The two-state data set of the porose octotaxic organs revealed multiple evolution of this character (Fig. 3C). This agrees with the ASR of the second data set (Fig. 3D; coded with the different types of porose organs) in which porose areas, saccules type1 and 2 do not share one most recent common ancestor (MRCA). More precisely, saccules type2 evolved two times and porose areas also at least twice. Particular types of porose organs are typically restricted to one phylogenetic lineage, but with some exceptions. In one family (Achipteriidae), two different types of porose organs are present. Whereas species of the genus *Achipteria *possess porose areas, the genus *Parachipteria *has saccules type1, which has been proposed to be the derived form [78]. Results of the ASR of the octotaxic porose organs are supported by BF comparison, which decisively rejected a monophylum Poronota (hypothesis 3, Table 2). Ancestral state reconstructions of the development of sclerits in poronotic nymphs indicated multiple independent origin of this trait (Fig. 4A). With *Phauloppia cf. lucorum *we only had one representative of the character state "nymphs with microsclerits", such that we focused specifically on species showing "nymphs with macrosclerits". According to the reconstruction of Mesquite (Fig. 4A), this trait could have evolved at least two times which is supported by the BF of the tested alternative hypothesis (hypothesis 4, Table 2). The ASR of the gastronotic cuticle structure of juveniles suggested that wrinkles in nymphs evolved independently at least two to maximum four times (Fig. 4B). Consistent with this observation, BF comparison rejected the hypothesis enforcing a monophyletic subgroup "Circumdehiscentiae with wrinkled nymphs" (hypothesis 5, Table 2). The results of the ASR of pteromorphs (Fig. 4C, Table 3) indicated that this structure evolved twice within the Apheredermata and the BF of the alternative hypothesis strongly discriminated against a monophyletic clade of species with pteromorphs.

## Discussion

### Molecular phylogeny

Bayesian inference of the phylogeny of the Circumdehiscentiae based on a combined data set of fragments of three nuclear genes revealed a tree topology similar to previous molecular studies [27,28], especially to the most recent phylogeny published by Maraun et al. [31] which is based on the 18S rDNA gene. In the latter study the statistical support for most important nodes is higher than in our case, but we note that taxon sampling differs slightly between Maraun et al.'s and our study. Splits within the Apheredermata are statistically not well supported in our phylogeny, which can be interpreted as a strong indication for a period of rapid cladogenesis at a certain time in the past. Comparing the 18S tree with our results in detail revealed a different placement of *Scapheremaeus cf. palustris *in both phylogenies. Whereas it is grouped with *Ameronothrus maculatus *and four specimens of the family Scutoverticidae in our phylogeny, it is placed with *Eremaeozetes sp. *as sister taxon to *Tectocepheus velatus *in the 18S tree, but we emphasize that Maraun et al.'s [31] data set did not include any representatives of the Scutoverticidae. Furthermore, our results show that there is no close relationship of *A. maculatus *and the genus *Podacarus auberti cf. occidentalis *as already supposed by Grandjean [79]. However, Weigmann and Schulte [80] unified the seven genera of originally three families (Ameronothridae, Podacaridae, Aquanothridae) into one single family Ameronothridae, a system accepted by Norton and Behan-Pelletier [34]. The placement of *S. cf. palustris *and *A. maculatus *within the Scutoverticidae renders not only this family but also the Cymbaeremaeidae and Ameronothridae paraphyletic. These results could certainly be caused by a strong bias of one single gene. However, the paraphyly of these three families is also supported in our single gene analyses (data not shown). The non-monophyly of Cymbaeremaeidae is also supported by the 18S rDNA gene [31] with *S. palustris *and *Cymbaeremaeus cymba *not clustering together. Whether the family Scutoverticidae should be split up or extended with additional taxa (*S. cf. palustris*, *A. maculatus *and maybe others) remains unclear until more species of the remaining genera and closely related families (e.g. Ameronothridae, Passalozetidae, Licneremaeidae) are included in a comprehensive phylogenetic study. However, our data, which are certainly not comprehensive enough to allow for a full revision of these problematic families but are sufficient to hint at some taxonomic inconsistencies, indicate that the family Scutoverticidae might be split up in "Scutoverticidae s.s.", so far including only species of *Scutovertex *and in "Scutoverticidae s.l." with the remaining taxa. The paraphyletic resolution of Ameronothridae, Cymbaeremaeidae and the genus *Achipteria *appears to be the result of a lack of adequate synapomorphic morphological characters, calling for further detailed investigations not only on a strictly morphological basis, but also including alternative approaches in a framework of integrative taxonomy [11].

### Ancestral state reconstruction

Ancestral state reconstructions and phylogenetic hypothesis testing indicate that none of Grandjean's [35] main traits is a good tool to classify the investigated taxa. However, regarding scalps and centrodorsal setae, the only disagreement to traditional taxonomic classifications is caused by the placement of *Arthrodamaeus *sp. which clusters within the Apheredermata (Figs. 3A-B), supported by BF comparison of alternative phylogenetic hypotheses (Table 2). Reconstruction of the character history of the octotaxic system in adults which is eponymous for the Poronota clearly suggested multiple evolution of this diagnostic character (Figs. 3C-D). The general model of the origin of saccules proposed by Grandjean [78,81] is that porose areas invaginated and formed a saccule having a lumen encircled by porose walls. Thus, porose areas in the octotaxic system would represent the plesiomorphic character state. Concerning the question whether the small porose areas or minute saccules in various Licneremaeoidea (e.g. *Licneremaeus, Scutovertex*) do either represent a numerical and size regression or the plesiomorphic state of the typical octotaxic system, Norton and Alberti [82] argued that the early evolution of porose organs started small because they are small, even minute in some Licneremaeoidea (a probably paraphyletic assemblage according to [82]) - e.g. Scutoverticidae, Dendroeremaeidae [83] (in our study called saccules type2) -, in their opinion the most early-derived group of Poronota. According to the numerical regression, it should be mentioned that the octotaxic system is often reduced to one, two or three pairs of organs. Among the Scutoverticidae, for example, no species is known to have the full complement of four pairs, leading to the hypothesis that four pairs do not represent the ancestral state for this family. Norton and Alberti [82] noted that the earliest homologue of the octotaxic system might be a single pair of dermal glands that evolved by gene-duplication or by modification of developmental controls to four pairs. Our results of the ancestral state reconstructions now suggested, that, whatever character coding [either two-stated (Fig. 3C) or organ-type specific (Fig. 3D)] is used, the octotaxic system evolved independently many times in parallel within the Poronota. Furthermore, there are no indications that either porose areas or saccules type2 are the plesiomorphic state of the porose organs as proposed by Grandjean [78,81] and Norton and Alberti [82], respectively. Concerning the weakly supported nodes within the Poronota (Fig. 2), one could hypothesize that porose areas still can be traced back to one MRCA, a hypothesis clearly rejected by our BF comparison of alternative phylogenetic hypotheses (hypothesis 3, Table 2). Saccules type2, present in *Scutovertex *and *Exochocepheus hungaricus*, was inferred to have evolved twice from an ancestor lacking porose organs (Fig. 3D). These results reject the general hypothesis that Poronota represent a natural, monophyletic subdivision, as already supposed by Grandjean [38]; he also stated that the presence or absence of these organs alone is not sufficient for a grouping into Pycno- or Poronota. A recent study [34] avoids the terms Pycnonota and Poronota, though it differentiates between pycnonotic and poronotic taxa without implying that these represent monophyletic groupings. Woas [84] suggested that secretory porose organs probably represent functional adaptations, potentially leading to a multiple independent evolution of this morphological character complex. Additionally, it must be noted that in many poronotic families and genera species can have various types of porose organs (see table 1 in [82]). For example, both porose areas and saccules (type1) are found among species of Achipteriidae (Table 1) or *Trichoribates *[85]. In this regard a potential parallel or convergent evolution of the octotaxic system should already have been a point of discussion in former time. However, considering the complexity of these paired organs located at the more or less same notogastral positions, it appears unlikely that these structures evolved independently multiple times, pointing to the need of further detailed investigations on these structures. Recently, Weigmann [86] reported on a different formation of octotaxic organs on the left and right side of the body of one single specimen and attributed this phenomenon to the differential action of regulatory genes.

In addition to the three main characters, Grandjean [35] used the morphology of nymphs and larvae for a classification within Circumdehiscentiae. He divided the Poronota into three types: 1) species with wrinkled nymphs; 2) species having nymphs with macrosclerits, and 3) species having nymphs with microsclerits. Furthermore, Grandjean [35,38] postulated that those taxa with wrinkled nymphs should form a monophyletic group regardless whether they are pycno- or poronotic (see Background) and that they might represent an intermediate group (he formulated it as "à cheval sur la limite" meaning "at the frontiers") between the pycnonotic Apheredermata and Poronota. This concerns taxa of the following 11 families: Podacaridae, Charassobatidae, Ameronothridae, Scutoverticidae, Tectocepheidae, Passalozetidae, Cymbaeremaeidae, Licneremaeidae, Achipteriidae, Tegoribatidae and Phenopelopidae (formerly Pelopsidae). Wauthy [37] followed this proposal and named the group "Circumdehiscentiae with wrinkled nymphs". However, our results revealed that the wrinkled nymphal cuticle structure evolved (and got lost) in parallel multiple times among Circumdehiscentiae. This clearly rejects the hypotheses of Grandjean [35,38] and Wauthy [37] that taxa with wrinkled nymphs are monophyletic. Ancestral state reconstructions also showed that the MRCA of Circumdehiscentiae had unwrinkled nymphs (Fig. 4B), thus rejecting the assumption of Norton and Behan-Pelletier [34] that wrinkles in nymphs - because of their occurrence in apheredermous and eupheredermous taxa - seem to be the plesiomorphic or even ancestral state in Circumdehiscentiae.

Pteromorphs only occur (with exception to the eupheredermous Microzetidae) in adult poronotic mites and Travé [87] postulated that the presence or absence of this trait might serve as differentiation criterion within the Apheredermata, a hypothesis rejected by our data (Fig. 4C; Table 2).

### Character evolution conflicting with current classification

Our reconstruction of ancestral states within the Circumdehiscentiae shows that some previously used diagnostic characters are problematic for taxon classification, despite previous efforts to clarify plesio- and apomorphies [86,88]. These problems mainly arise from the difficulty to evaluate missing characters as reduced or never developed. However, specific traits could be still of appreciable value such as the presence of nymphal scalps or centrodorsal setae. Scalp retention is often correlated with the nature of dehiscence [35], which goes up after a striking process in immature instars. If the metamorphosis fails, for example due to a genetic defect, the molting individual would stall and die. Furthermore, the absence of centrodorsal setae correlates with the scalps [35] because species retaining scalps on their notogaster are dorsodeficient (have lost setae *da*, *dm*, *dp*). Thus, dorsodeficient nymphs correspond to the Eupheredermata and integridorsal nymphs to the Apheredermata, respectively. As an exception the two dorsodeficient apheredermous families Ceratoppiidae and Liacaridae group with the Eupheredermata with dorsodeficient nymphs, thus suggesting a possible loss of scalps in nymphal stages and adults.

Altogether, none of Grandjean's main traits can be used as diagnostic character for classification of the Circumdehiscentiae, because of clear evidence for multiple parallel evolution (and in some cases also losses) of these traits. In the case of the octotaxic system this could be argued with a possible correlation of secretory notogastral porose organs and ecology. Norton and Alberti [82] still noted that poronotic mites with modified porose areas (saccules, multiplications) inhabit non-soil microhabitats (e.g. mosses and lichens on rocks or trees) and therefore internalization may reduce water-loss through porose surface. But they finally stated that there is no biological or ecological correlation that would help to understand frequent convergent evolution. We hypothesize that maybe there is no correlation of the secretory organs with the present ecology. Considering the old age of Oribatida - the crown radiation of Apheredermata took place at the Triassic/Jurassic boundary (c. 200 MYA) [19,89] or according to Schaefer et al. [90] in the Permian - it might be more adequate to search for congruent environmental conditions in the past, when the explosive radiation within the Apheredermata took place. A monophylum "Circumdehiscentiae with wrinkled nymphs" was clearly rejected by the results of the ancestral state reconstructions, implying that the wrinkled nymphal cuticle structure is of little value for classification. The potential function of these wrinkles was already well studied by Smrž [91] for some oribatid taxa. Thereby he recovered two different types of wrinkling (e.g. *Hermannia gibba *versus *Scutovertex minutus*) and a potential correlation between environmental conditions and type of wrinkling.

Typically, studies on character evolution focus on either the potential advantages of a trait in a given environment [92-94] or mechanisms that create novel phenotypes [95,96]. However, Wiens et al. [97] stated that these investigations may be necessary to elucidate why a trait has evolved in a particular instance but not why it has evolved multiple times. According to Wiens et al. [97] at least two additional factors are important in determining the number of origins, namely the biogeographic context of the selective environment and competitive interactions. The first point means that a trait which is adapted to a selective environment may evolve multiple times in geographically isolated regions with identical selective environments (e.g. [98]). We hypothesize that this might be a possible reason for the multiple parallel evolution of the octotaxic system and wrinkled gastronotic cuticle structure in nymphs, but further detailed correlation studies including more taxa are necessary to allow for robust conclusions.

## Conclusions

Ancestral state reconstructions of six diagnostic characters revealed some conflicts to the current morphological classification within the oribatid mite group Circumdehiscentiae. Most of these presumed diagnostic, in particular the octotaxic system (eponymous for the subdivision "Poronota") and the wrinkled gastronotic cuticle of nymphs (taxa having these nymphs were hypothesized to be monophyletic within "poronotic" mites) were inferred to have evolved/been lost multiple times independently, subsequent to an explosive radiation of the "Higher Oribatida" into its major lineages. One likely reason for the parallel or convergent evolution of particular traits might be based on the biogeographic context of the selective environment, meaning that evolution produced similar phenotypes in different geographically isolated habitats [97]. Elucidating the exact (genetic) mechanisms responsible for the observed multiple origin of particular traits and character states remain a task for the future. In particular we want to note that at present we cannot decide whether the genetic basis for these traits evolved several times independently or whether this patchy distribution of traits along a phylogeny is due to atavisms, the preservation of previously existing phenotypic features in the genome, but not expressed in the ancestors. Regardless of the exact mechanism responsible for the observed patterns, the investigated traits do not reflect the phylogenetic relationships among circumdehiscent mites.

Thus, to conclude, the present study clearly shows that the current classification schemes of the Circumdehiscentiae are inappropriate. In our opinion, the most recent proposal by Norton and Behan-Pelletier [34] with 24 superfamilies and no higher groupings best reflects the taxonomic situation/uncertainty within the "Higher Oribatida". However, for future prospects to clarify the taxonomy of the circumdehiscent mites an integrative approach based on various sources of evidence, including molecular data [11], and an increased taxon sampling seems necessary.

## Authors' contributions

SS, TP and GK designed the study. SS performed molecular genetic lab work and together with SK analyses and interpretation of data. TP carried out morphological investigations. SS drafted the manuscript. SK, CS and GK critically revised the manuscript. All authors read and approved the final manuscript.
